# Comparison of Three CD3-Specific Separation Methods Leading to Labeled and Label-Free T Cells

**DOI:** 10.3390/cells10112824

**Published:** 2021-10-21

**Authors:** Ronald Weiss, Wilhelm Gerdes, Rommy Berthold, Ulrich Sack, Ulrike Koehl, Sunna Hauschildt, Anja Grahnert

**Affiliations:** 1 Institute of Clinical Immunology, Medical Faculty, Leipzig University, Johannisallee 30, 04103 Leipzig, Germany; ronald.weiss@uni-leipzig.de (R.W.); sack@uni-leipzig.de (U.S.); Ulrike.Koehl@medizin.uni-leipzig.de (U.K.); 2 Cell.Copedia GmbH, Bosestrasse 4, 04109 Leipzig, Germany; gerdes@cellcopedia.com (W.G.); berthold@cellcopedia.com (R.B.); 3 Fraunhofer Institute for Cell Therapy and Immunology, Perlickstraße 1, 04103 Leipzig, Germany; 4 Institute of Cellular Therapeutics, Hannover Medical School, Feodor-Lynen-Str. 21, 30625 Hannover, Germany; 5 Institute of Biology, Faculty of Life Sciences, Leipzig University, Talstrasse 33, 04103 Leipzig, Germany; shaus@rz.uni-leipzig.de

**Keywords:** TACS^®^, MACS^®^, REAlease^®^, separation, isolation, T cells, CD3, proliferation

## Abstract

T cells are an essential part of the immune system. They determine the specificity of the immune response to foreign substances and, thus, help to protect the body from infections and cancer. Recently, T cells have gained much attention as promising tools in adoptive T cell transfer for cancer treatment. However, it is crucial not only for medical purposes but also for research to obtain T cells in large quantities, of high purity and functionality. To fulfill these criteria, efficient and robust isolation methods are needed. We used three different isolation methods to separate CD3-specific T cells from leukocyte concentrates (buffy coats) and Ficoll purified PBMCs. To catch the target cells, the Traceless Affinity Cell Selection (TACS^®^) method, based on immune affinity chromatography, uses CD-specific low affinity Fab-fragments; while the classical Magnetic Activated Cell Sorting (MACS^®^) method relies on magnetic beads coated with specific high affinity monoclonal antibodies. The REAlease^®^ system also works with magnetic beads but, in contrast to MACS^®^, low-affinity antibody fragments are used. The target cells separated by TACS^®^ and REAlease^®^ are “label-free”, while cells isolated by MACS^®^ still carry the cell specific label. The time required to isolate T cells from buffy coat by TACS^®^ and MACS^®^ amounted to 90 min and 50 min, respectively, while it took 150 min to isolate T cells from PBMCs by TACS^®^ and 110 min by REAlease^®^. All methods used are well suited to obtain T cells in large quantities of high viability (>92%) and purity (>98%). Only the median CD4:CD8 ratio of approximately 6.8 after REAlease^®^ separation differed greatly from the physiological conditions. MACS^®^ separation was found to induce proliferation and cytokine secretion. However, independent of the isolation methods used, stimulation of T cells by anti CD3/CD28 resulted in similar rates of proliferation and cytokine production, verifying the functional activity of the isolated cells.

## 1. Introduction

Identification and characterization of specific cell populations are of great importance for research, diagnostic, and therapeutic purposes. To optimize the characterization of such populations the cells have to be devoid of contaminating cells. Hence, reliable adequate isolation methods are required to separate the cells of interest. 

Here, we compare three positive isolation methods to obtain CD3-positive T cells. These cells play an important role in adoptive immune responses against pathogens, allergens, and cancer cells and, thus, they constitute an integral part of diagnosis. Much attention has been paid to these cells lately because of their promising role in the treatment of cancer by adoptive cell transfer. 

In view of these successful applications, it is essential that specific efficient and robust cell separation methods are available to obtain pure and functional cells. 

One of the methods used here to isolate T cells is based on a classical positive selection by antibody-dependent Magnetic Activated Cell Sorting (MACS^®^). The heterogeneous cell suspension is mixed with magnetic beads that carry an antigen specific capture antibody (e.g., against CD3) that connects the beads with the cells of interest. When loaded onto a column containing a ferromagnetic matrix in a magnetic field, the magnetic bead-labeled cells remain in the column while the unlabeled cells pass. After removing the magnetic field, the remaining population representing the cells of interest can be eluted [1,2].

Another method we used is the fragment antigen-binding (Fab) (antibody fragment) Traceless Affinity Cell Selection (TACS^®^) based on immunoaffinity chromatography. Antigen-specific strep-tagged Fab-fragments are reversibly bound to an agarose matrix via Strep-Tactin^®^ [3]. The heterogeneous cell suspension is loaded onto the Fab-labeled agarose matrix, where target cells are captured by clusters of antigen-specific Fab fragments with high avidity, while non-target cells pass through the matrix. To elute the target cells, the binding of the Fab-fragments to the agarose matrix will be destroyed due to the addition of D-biotin. This not only results in the disruption of the high avidity Fab-fragment clusters but also leads to a dissociation of the Fab-fragments from the cells and, thus, label-free target cells [3,4].

As a third method, we used the REAlease^®^ technology, a special type of MACS^®^ that works similar to TACS^®^ with antigen-specific, low-affinity antibody fragments instead of whole antibodies. In the case of REAlease^®^, the antibody-fragments are reversibly connected to magnetic beads. Following the classical above-mentioned MACS^®^ procedure, the binding between cells and magnetic beads can be disrupted by a release solution, resulting in the dissociation of the antibody fragments and, consequently, in the generation of label-free cells [5].

In this study, we compared three different positive isolation methods to separate CD3-specific T cells from buffy coats (MACS^®^, TACS^®^) and PBMCs (TACS^®^, REAlease^®^) and analyzed yield, viability, purity, and functional activity of the separated cells.

## 2. Materials and Methods

### 2.1. Cell Separation

**Figure 1:**
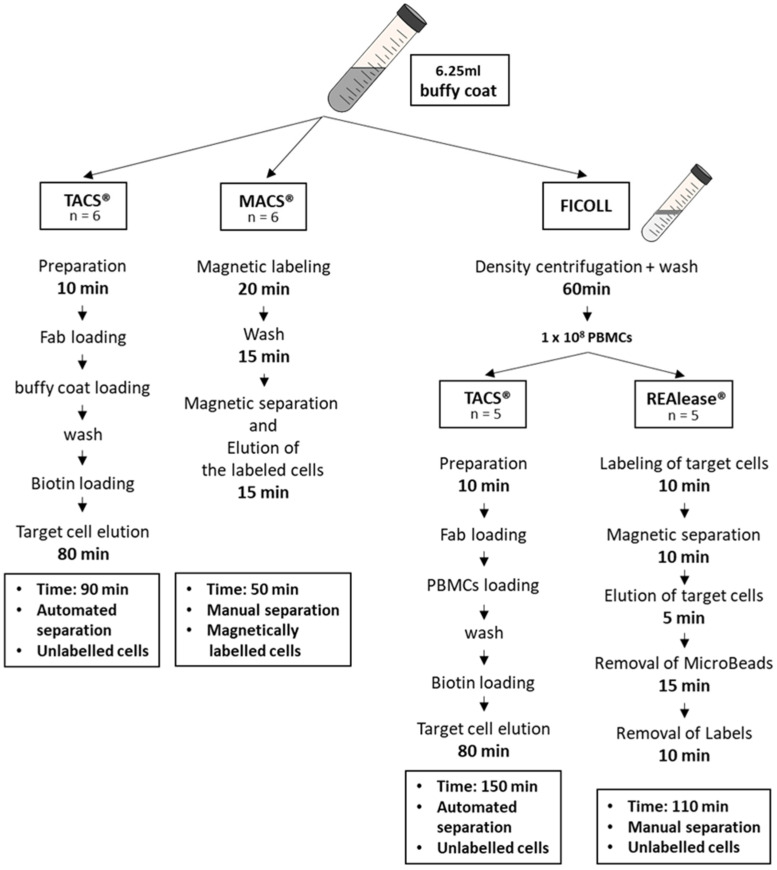
Overview of the isolation protocols. CD3-positive cells were either isolated from buffy coats using the MACS^®^ and TACS^®^ technology or from buffy coat-derived PBMCs using the TACS^®^ and REAlease^®^ system. Shown are the different procedures used to isolate CD3-positive T cells.

To compare the different isolation methods for CD3-positive cells, we used the MACS^®^ and TACS^®^ system to directly isolate the cells from buffy coats and the TACS^®^ and REAlease^®^ system for the isolation of CD3-positive cells from Ficoll purified PBMCs (Figure 1).

### 2.2. Cell Separation from Buffy Coat Using MACS^®^ and TACS^®^ System

Cells from healthy donors were isolated from buffy coats acquired from the blood service (Institute of Transfusion Medicine, University Hospital Leipzig; ethics license 272-12-13082012). Before applying the MACS^®^ and the TACS^®^ systems, 6.25 mL buffy coats were diluted with 6.25 mL cell isolation buffer (PBS/1 mM EDTA + 0.5% BSA), respectively.

To isolate CD3-positive cells by MACS^®^, the StraightFrom™ Whole Blood CD3 MicroBeads and Whole Blood Column Kit (both from Miltenyi Biotech, Bergisch Gladbach Germany) were used according to the manufacturer’s protocol.

Isolation of CD3-positive cells by TACS^®^ was carried out with the CD3 Isolation Kit for FABian^®^ (IBA GmbH, Göttingen, Germany) according to the manufacturer’s protocol. Automated cell separation was performed with FABian^®^ (Cell.Copedia, Leipzig, Germany), with the available standard program. 

### 2.3. Cell Separation from PBMCs Using TACS^®^ and REAlease^®^ System

Human peripheral blood mononuclear cells (PBMCs) from buffy coats (healthy donors) were obtained by Ficoll-Paque^TM^ Plus (GE Healthcare, Solingen, Germany) density centrifugation. After repeated washing in PBS containing 0.3 mM EDTA, the CD3-positive cells were isolated from 1 × 10^8^ PBMCs by the FABian^®^ (Cell.Copedia, Leipzig, Germany) based automated CD3 TACS^®^ technology (IBA GmbH, Göttingen, Germany) and the CD3 MicroBead REAlease^®^ technology (Miltenyi Biotech, Bergisch Gladbach, Germany), according to the manufacturer’s protocol. Both the TACS^®^ and REAlease^®^ separations will lead to label free-cells (Supplementary Figure S1 and Supplementary Material and Methods).

Following all separations, cells were treated with Tuerks solution (Merck, Darmstadt, Germany) to lyse erythrocytes and were counted using a Neubauer chamber.

### 2.4. Cell Proliferation Assay

Cell proliferation assays were carried out with Ficoll purified PBMCs as well as CD3-positive cells obtained using the different isolation methods. CD3-positive cells (5 × 10^5^/500 µL) were cultured in X-VIVO 10™ media (Lonza Group Ltd., Basel, Switzerland) supplemented with 2% AB serum (Blood Service, Institute of Transfusion Medicine, University Hospital Leipzig, Leipzig, Germany), 100 U/mL IL-2 (PeproTech, Hamburg, Germany), in 48-well microtiter plates (Thermo Fisher Scientific, Waltham, MA, USA). The physiological activation of CD3-positive cells was achieved by the addition of the Dynabeads™ Human T-Activator CD3/CD28 (anti CD3/CD28, Thermo Fisher Scientific, Waltham, MA, USA) at a bead-to-cell ratio of 3:1. Thereafter, the cells were incubated for 6 days at 37 °C in a humidified atmosphere with 5% CO_2_.

#### 2.4.1. Microscopy-Based Proliferation Assay

Cell morphology and proliferation were visualized with a Nikon Eclipse TE 2000-E microscope using the NIS-Elements F 3.2 software (Nikon, Tokyo, Japan).

#### 2.4.2. VPD450-Based Proliferation Assay

Proliferation capacity was also monitored by cell division-dependent decrease of Violet Proliferation Dye 450 (VPD450, Becton Dickinson, Heidelberg, Germany), staining intensity by flow cytometry. Therefore, 1 × 10^7^/mL CD3-positive cells were labeled with 1 µM VPD450 and incubated for 15 min at 37 °C. After three washing steps, cells were resuspended in complete medium and proceeded to cell culture. VPD450 intensity was measured after 6 days in the presence and absence of Dynabeads™ Human T-Activator CD3/CD28 (Thermo Fisher Scientific, Waltham, MA, USA) by a FACSCanto II flow cytometer (Becton Dickinson, Heidelberg, Germany). 

### 2.5. Detection of Cytokines

CD3-positive cells (1 × 10^6^/mL) were activated by the addition of the Dynabeads™ Human T-Activator CD3/CD28 (Thermo Fisher Scientific, Waltham, MA, USA) at a bead-to-cell ratio of 3:1. After 6 days, IFNγ, IL-4, and IL-10 concentrations in culture supernatants were determined using an IFNγ-, IL-4-, and IL-10-ELISA kit (PeproTech, Hamburg, Germany) and IL-17A with an IL-17A-ELISA MAX™ Deluxe Set (BioLegend, San Diego, CA, USA) according to the manufacturer’s protocol. 

### 2.6. Annexin-V Assay

The effect of the different cell separation methods on cell viability was detected by using the FITC Annexin V Apoptosis Detection Kit with PI (BioLegend, San Diego, CA, USA) according to the manufacturer’s protocol. Briefly, 2 × 10^5^ cells were washed with Annexin binding buffer and resuspended in 100 μL of Annexin-V and PI dual-stain solution (0.1 μg of Annexin-V FITC and 1 μg of PI) for 15 min in the dark. After adding Annexin binding buffer, cells were analyzed by a FACSCanto II flow cytometer (Becton Dickinson, Heidelberg, Germany). 

### 2.7. Cell Viability and Receptor Detection

Cell viability was analyzed by incubating 1 × 10^5^ cells in the presence of Fixable Viability Dye FVD eFlour^®^ 780 (1:500; Thermo Fisher Scientific, Waltham, MA, USA) for 25 min at 4 °C. After washing three times with 3% FCS in PBS, cell surface molecules were detected by the following antibodies:

- Whole mononuclear white cell population: aCD45-PerCP (2D1; BioLegend, San Diego, CA, USA), aCD3-V450 (UCHT1; Becton Dickinson, Heidelberg, Germany), aCD19-PE (SJ25C1; Becton Dickinson, Heidelberg, Germany), aCD14-APC (M5E2; BioLegend, San Diego, CA, USA), aCD16-FITC (B73.1; BioLegend, San Diego, CA, USA), and aCD56-FITC (HCD56; BioLegend, San Diego, CA, USA).
- Erythrocyte contamination: aCD235a-FITC (HI264; BioLegend, San Diego, CA, USA), aCD45-PerCP (2D1; BioLegend, San Diego, CA, USA), and aCD81-APC (1D6; Thermo Fisher Scientific, Waltham, MA, USA).
- T cell markers: aCD3-V450 (UCHT1, Becton Dickinson, Heidelberg, Germany), aCD4-PE (OKT4; BioLegend, San Diego, CA, USA), aCD8-FITC (SK1; BioLegend, San Diego, CA, USA), and aCD45-PerCP (2D1; BioLegend, San Diego, CA, USA).
- Proliferation assay: aCD3-FITC (SK7; BioLegend, San Diego, CA, USA), aCD8-APC (SK1; BioLegend, San Diego, CA, USA).

Cells were incubated with the antibodies for 20 min at 4 °C in the dark. After washing three times (PBS + 10% Emagel (Pirmal Healthcare, Northumberland, UK) + 0.1% NaN_3_) the cells were fixed (1% formaldehyde) and analyzed by a FACSCanto II flow cytometer (Becton Dickinson, Heidelberg, Germany). 

Flow cytometry data analysis was performed with the FACS Diva software 8.0.1 (Becton Dickinson, Heidelberg, Germany). Data analysis for histogram overlays were performed with FlowJo 10.7.1 (FlowJo, Ashland, OR, USA). Gating strategies are given in Supplementary Figures S2–S5.

## 3. Results

### 3.1. Yield and Viability of CD3-Positive Cells

**Figure 2:**
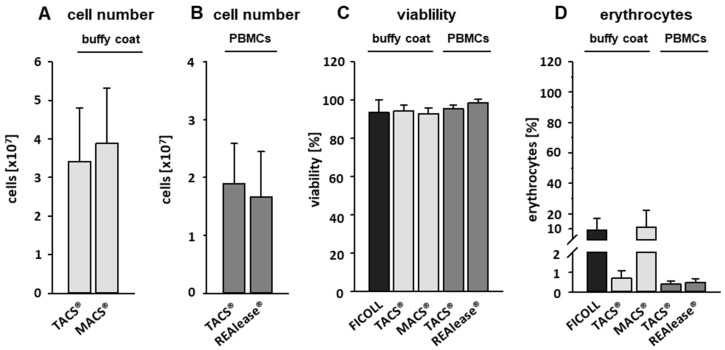
Cell number, viability, and erythrocyte contamination. CD3-positive cells were either separated from buffy coats by the MACS^®^ and TACS^®^ system or from PBMCs by the TACS^®^ and REAlease^®^ system. Cell numbers (**A**,**B**) were determined by manual counting in a Neubauer chamber. Shown are the mean of cell number ± SD, *n* = 6 (buffy coat) and *n* = 5 (PBMCs). The viability (**C**) was measured by FITC Annexin V Apoptosis Detection Kit with PI and contamination with erythrocytes (**D**) by measuring CD235a expression via flow cytometry. Shown are the percentages of viable cells negative for FITC Annexin V and PI (**C**) or positive for CD235a (**D**) ± SD, *n* = 6.

To compare the three isolation methods used to isolate CD3-positive cells, different protocols were applied. Cells were either directly isolated from buffy coats (TACS^®^ and MACS^®^) or from PBMCs (TACS^®^ and REAlease^®^). PBMCs were obtained from 6.25 mL buffy coat by Ficoll-Paque^TM^ Plus density centrifugation (Figure 1). According to the manufacturer’s protocols, the isolation of T cells by TACS^®^ and MACS^®^ directly from buffy coats took 90 min and 50 min, respectively. Due to the previous Ficoll enrichment of PBMCs, the total isolation time of T cells from PBMCs by TACS^®^ and REAlease^®^ amounted 150 min and 110 min, respectively. Independent of the CD3 separation method applied, the yield did not differ very much (Figure 2A,B). Considering that approx. 50% of the 1 × 10^8^ used PBMCs are CD3-positive lymphocytes (~5 × 10^7^) the recovery amounted to about 35% (Figure 2B). Further, a cell viability as high as 95% was reached by all methods tested (Figure 2C). As shown in Figure 2D, cell isolation from buffy coats resulted in a high erythrocyte contamination after Ficoll and MACS^®^ separation (approx. 10%) while the contamination was <1% after TACS^®^ and REAlease^®^ separation. 

### 3.2. Purity and Characterization of CD3 Isolated Cells

**Figure 3:**
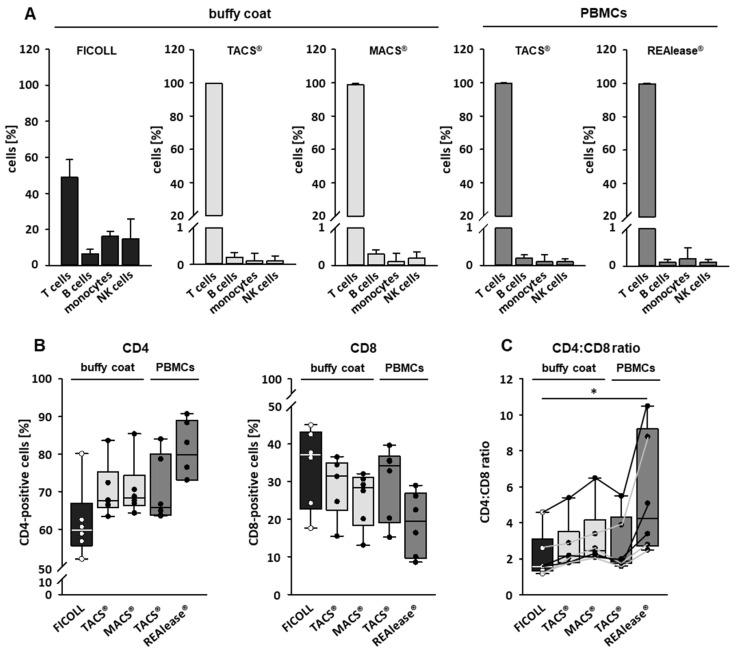
Purity of separated CD3-positive cells. The purity of the cells after isolation was determined by flow cytometry. CD3-positive cells were either separated from buffy coats by the MACS^®^ and TACS^®^ system or from Ficoll purified PBMCs by the TACS^®^ and REAlease^®^ system. Data show the percentage of isolated cells ± SD, *n* = 6 (**A**). CD4-positive and CD8-positive cells within the CD3-positive cell population were determined by flow cytometry (**B**) and the CD4:CD8 ratio was calculated (**C**). The lines in (**C**) connect data derived from the individual donors, *n* = 6. Box plots indicate median (horizontal lines), interquartile range (box), and range (whiskers). Mann Whitney Rank Sum Test, * = *p* ≤ 0.05.

Isolation of cells by specific CD markers is a reliable method to enrich the cell type of interest and to eliminate contaminating cells. To analyze the purity and contamination of the CD3 isolated cells we used flow cytometry and defined following cells by their specific markers: T cells (CD3-pos.), B cells (CD19-pos.), monocytes (CD14-pos.), and NK cells (CD16/56-pos.). As shown in Figure 3A, about 50% of buffy coat derived PBMCs by Ficoll separation are T cells, while B cells, monocytes, and NK cells make up 7, 16, and 15% of the total population, respectively. After separation, the purity of T cells was higher than 98% irrespective of the separation method and cell source used. Contaminations with B cells, monocytes, and NK cells were mostly far below 0.5% (Figure 3A). 

We further characterized the CD3-positive cells by identifying T-helper cells (CD4-pos.) and cytotoxic T cells (CD8-pos.) in the T cell fraction by flow cytometry. As shown in Figure 3B, all cell isolation procedures resulted in an increase of CD4-positive and a decrease of CD8-positive fractions compared to Ficoll-isolated PBMCs. The changes were most prominent when cells were separated by REAlease^®^. This different distribution of the two T cell populations is also mirrored by a moderate (TACS^®^ and MACS^®^ separations) or a strong increase (REAlease^®^ isolation) of CD4/CD8 ratios (Figure 3C). 

### 3.3. Functional Activity of CD3 Isolated Cells

Besides purity, functionality of isolated cells is another important factor when assessing isolation methods. Here, we evaluated the functionality of isolated activated T cells by determining the proliferation capacity and cytokine production, both important biological activities that are upregulated when T cells provoke an effective immune response. 

**Figure 4:**
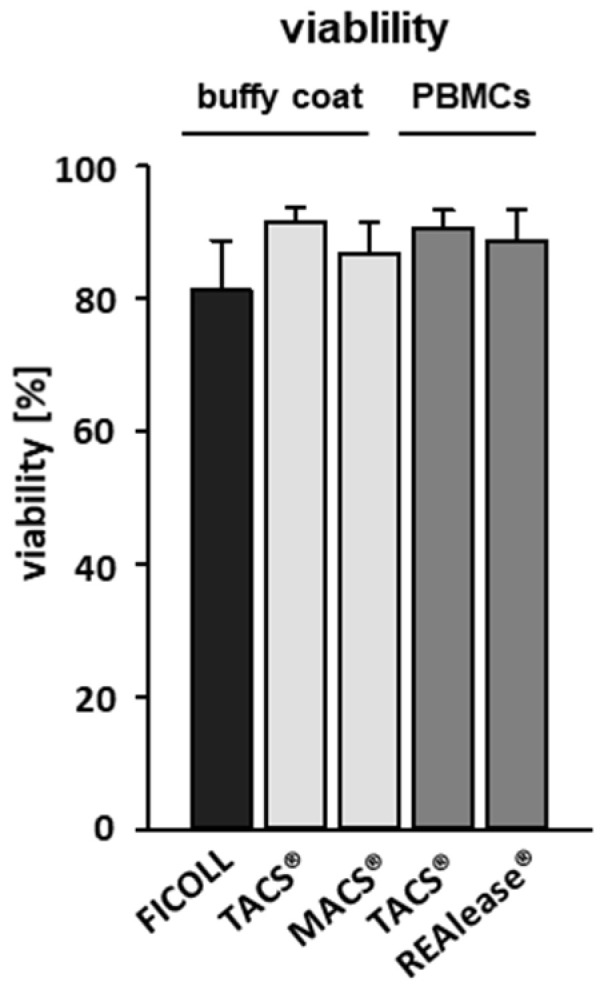
Effect of the different CD3-dependent isolation methods on cell viability. CD3-positive cells were either separated from buffy coats by the MACS^®^ and TACS^®^ system or from Ficoll purified PBMCs by the TACS^®^ and REAlease^®^ system. After separation, cells were cultured (1 × 10^6^/mL) for six days in medium. The viability of the cells was determined via flow cytometry by using Fixable Viability Dye FVD eFlour^®^ 780 after incubation. Shown are the percentages of viable cells negative for FVD eFlour^®^ 780 ± SD, *n* = 4.

To determine proliferation rates, we performed flow cytometry analysis based on VPD450 (proliferation) and FVD eFlour^®^ 780 (viability) staining 6 days after stimulation with anti CD3/CD28 beads (aCD3/CD28). We could show that the viability of unstimulated T cells 6 days after separation by all CD3-specific methods was comparatively high (>85%) while slightly lower values (80%) were reached by Ficoll-separated PBMCs (Figure 4).

**Figure 5:**
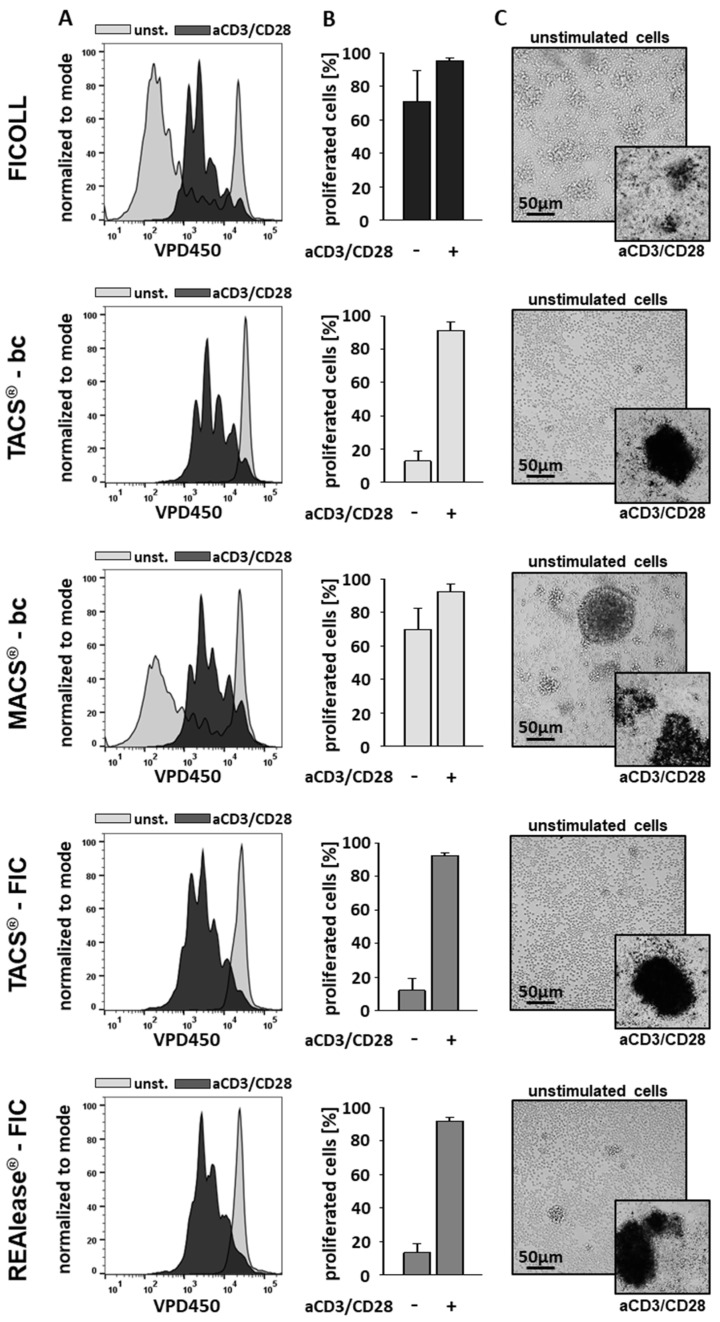
Effect of the different CD3−dependent isolation methods on cell morphology and proliferation. After separation, cells were labeled with VPD450 and cultured (1 × 10^6^/mL) in the presence or absence of Dynabeads™ Human T-Activator CD3/CD28 at a bead-to-cell ratio of 3:1 (anti CD3/CD28 stimulation (aCD3/CD28)). After 6 days the lymphocyte proliferation was analyzed by flow cytometry. Representative histogram overlays show the discrimination strategy between proliferating (aCD3/CD28) and non-proliferating (unst.) lymphocytes (**A**). The results (*n* = 4) are represented as the mean ± SD of the proliferating CD3-positive lymphocytes (**B**). The morphology of the stimulated (aCD3/CD28) and unstimulated cells was examined using brightfield microscopy (**C**). Shown is one representative experiment out of four.

Stimulation with aCD3/CD28 beads resulted in high proliferation rates of CD3-positive T cells independent of the isolation method used (Figure 5A,B). Whereas spontaneous proliferation of unstimulated cells separated by TACS^®^ and REAlease^®^ were low, the use of the Ficoll and MACS^®^ separation technique led to high proliferative activities. These findings agree well with cluster formation in unstimulated cells, which is shown here by microscopy (Figure 5C). Thus, the elevated proliferation rates and the increased cluster formation in unstimulated controls indicate that the cells had been pre-activated.

**Figure 6:**
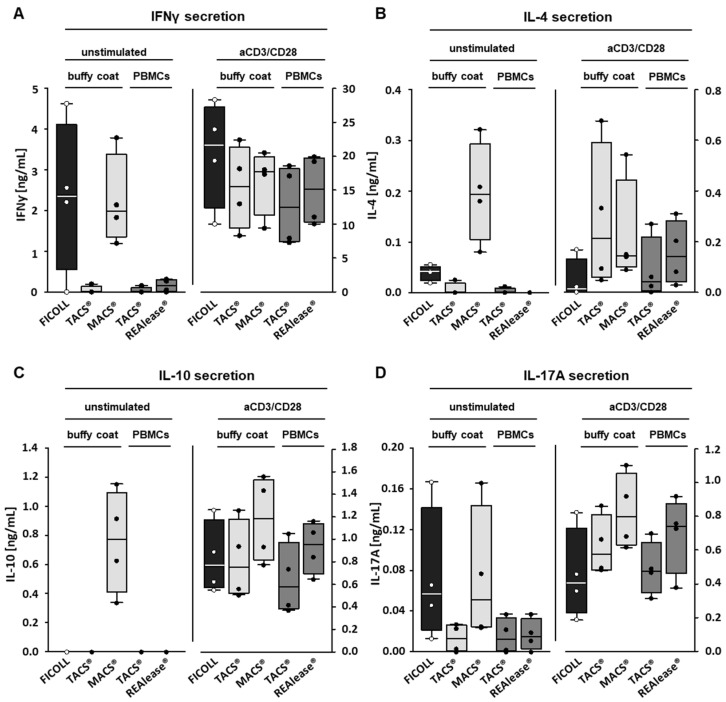
Effect of the different CD3-dependent isolation methods on cytokine production. To determine the effect of the different isolation methods on the production of cytokines induced by CD3-positive cells, the VPD450-labeled cells were cultured (1 × 10^6^/mL) in the presence of medium or with Dynabeads™ Human T-Activator CD3/CD28 at a bead-to-cell ratio of 3:1 (anti CD3/CD28 stimulation (aCD3/CD28)). After six days, IFNγ (**A**), IL-4 (**B**), IL-10 (**C**), and IL-17A (**D**) concentrations were determined by ELISA, *n*= 4. Box plots indicate median (horizontal lines), interquartile range (box), and range (whiskers).

Besides proliferation, we measured aCD3/CD28 induced cytokine production, another means to determine functional activity. As seen in Figure 6A–D, stimulation of T cells with aCD3/CD28 beads results in an upregulation of the cytokines IFNγ, IL-4, IL-10, and IL-17A, while resting T cells hardly produced any or minor amounts of these cytokines. However, after separation by MACS^®^ or Ficoll, all four cytokines or IFNγ and IL-17A, respectively, were found to be elevated in the absence of a stimulation. These data, which agree well with the increased proliferation rates, confirm that the cells have been pre-activated during the separation procedure.

## 4. Discussion

T cells, important cells of the adaptive immunity, are involved in the establishment and maintenance of the immune system’s response, homeostasis, and memory. Besides other immune cells, they are particularly important to fight pathogens, allergens, and cancer [6]. They are of great clinical relevance, especially since adoptive cell transfer (ACT), which implies transplantation of allogenic or autologous immune cells, has become a promising tool in cancer therapy. Some of the ACT approaches simply rely on expanding cell numbers ex vivo, while others are based on genetically engineered immune cells [7]. This latter technique has been successfully applied when generating T cells that express chimeric antigen receptor (CAR) constructs, which, by binding to the respective antigen, are able to destroy cancer cells. The use of T cells isolated by CD3 via MACS^®^ and TACS^®^, two of the here described isolation methods, lead to functional aCD19-CAR T cells in experimental settings, as shown recently [3,8]. Currently four aCD19-CAR T cell products (Yescarta^®^, Tecartus^®^, Kymriah^®^, Breyanzi^®^) are approved by EMA and/or FDA to fight B cell lymphomas and leukaemias [9,10]. The T cells used are either separated by CD3-specific separation methods (Kymriah^®^) [11], restricted to CD4-positive and CD8-positive T cells (Tecartus^®^, Breyanzi^®^) [12,13], or not specifically separated any further after leukapheresis (Yescarta^®^) [14]. Especially when producing CAR T cells for clinical use it is of great importance to deal with pure T cell fractions. It has been shown that contaminating leukaemic B cells can also be transduced with the CAR construct, so that binding in cis of the B cell CAR to the B cell marker CD19 will mask the aCD19-CAR T cell target and, thus, prevent T cells from killing the leukaemic B cells [15]. We found, when isolating CD3-positive cells by the three different methods and comparing their effect on viability, purity, and functionality, that the contamination with B cells and also with NK cells and monocytes was very low, independent of the CD3 separation method used; important information when purity of T cells matters as for chimerism analysis. Chimerism analysis after allogeneic haematopoietic stem cell transplantation by PCR methods can help to detect a relapse of the disease or of graft failure at an early time point and analyses of engraftment of lineage specific cell populations, e.g., of haematopoietic progenitor cells (CD34-positive) or T cells (CD3-positive), can give more sensitive information regarding graft rejection or disease relapse [16,17,18]. 

T cell populations can be subdivided into CD8-positive cytotoxic T lymphocytes (CTL) and CD4-positive T helper cells (Th), with the latter consisting of further subpopulations (e.g., regulatory T cells (Tregs), Th1, Th2, Th17), which all have different functions [19]. Should T cells be required whose function rely on a specific CD4:CD8 ratio, as has been described for CAR-T cells [20,21], the data presented here are very helpful. They show that the CD4:CD8 ratio of T cells separated by REAlease^®^ had clearly shifted to CD4 compared to the ratio of the two subpopulations, which after isolation by TACS^®^, MACS^®^, and Ficoll was within the physiological range of 2 to 3. This ratio agrees well with published in vivo data [22,23]. However, in order to obtain a defined CD4:CD8 ratio, the CD4 and CD8 T cell subsets must be separated by using CD4- and CD8-specific isolation methods [24], which bypass isolation of CD3 T cells before beginning the CAR T cell production [25].

The here described separation methods are based on positive selection via the recognition of the T cell antigen CD3. CD3 as part of the T cell receptor (TCR) complex is involved in signaling mechanisms that result in the activation of T cells [26,27]. After recognition of antigens presented by other immune cells, T cells secrete cytokines and proliferate to induce an effective immune response [28,29]. When activating T cells with aCD3/CD28 beads, all cells, regardless of the isolation method used, showed a higher potential to proliferate and secrete cytokines than the unstimulated controls, indicating that the isolated cells are capable of exerting specific immune functions. As aCD3/CD28 bead stimulation is non-specific, all T cells including the subtypes are activated and, thus, the resulting cytokine profile is rather heterogeneous; consisting of both pro-inflammatory and anti-inflammatory mediators. While IFNγ is mainly produced by CTLs [30] and Th1 cells; IL-4, IL-10, and IL-17A are signature cytokines of Th2, Tregs, and Th17 cells, respectively [19]. It cannot be excluded that separation techniques based on whole antibodies that show high affinity and strong binding capacity to the target antigen (e.g., clone OKT3 and anti-Leu4 [31]) contribute to the activation of T cells. As activation mostly occurs via the TCR complex, a strong binding of antibodies to CD3 can lead to an activation/pre-stimulation of the T cells already during the isolation procedure. This very likely occurred when using the MACS^®^ separation method. Should this happen, a negative selection is useful. However, negative selections may result in a lower purity of the target cells [32] and need pre-separation of PBMCs. As an alternative to circumvent pre-activation of the cells and to obtain cells of high purity and functionality, isolation by TACS^®^ and REAlease^®^ can be used. These two methods not only rely on low affinity binding of Ab fragment to the antigen, but also on their dissociation of the fragments from the antigen, leading to label free cells (Supplementary Figure S1 and Supplementary Material and Methods). This has the advantage that all epitopes of the target protein CD3 are available and not occupied by an antibody after separation. As a result, the free binding sites can be used to determine T cells via the CD3 molecule [33]. In line with these data, T-helper cells isolated by CD4-REAlease^®^ were reported to be suitable for electrophysiology and ion channel studies [34] and we showed previously that CD14-TACS^®^ separation of monocytes yielded intact cells that can be used to generate functional dendritic cells [4]. 

The here described pre-activation of the cells separated by Ficoll (w/o antibodies) is very likely caused by other immune cells (e.g., monocytes), which are present in the cell culture. 

Taken together we here show that all three separation methods are equally suited to obtain T cells of high purity, viability, and activity. The isolation by TACS^®^ and REAlease^®^ seems to be more appropriate when pre-activation and labeling of cells matter. Should a direct separation from whole blood or a physiological CD4:CD8 ratio be required then TACS^®^ and MACS^®^ are the methods of choice. Independent of the separation method used, all T cells show similar proliferation rates and functional activity.

## Data Availability

The datasets generated and analyzed during the current study are available from the corresponding author on reasonable request.

## Supplementary Materials

The following are available online at https://www.mdpi.com/article/10.3390/cells10112824/s1, Material and Methods: Determination of label free-cells, Figure S1: Determination of label free-cells, Figure S2: Gating strategy to determine erythrocyte contamination, Figure S3: Gating strategy to determine the mononuclear white cell population, Figure S4: Gating strategy to determine the CD4 and CD8 T cell subsets, Figure S5: Gating strategy to determine proliferation of T cells.
