# Supplementary material for: Comparison of Three CD3-Specific Separation Methods Leading to Labeled and Label-Free T Cells
Source: Cells. 2021 Oct 21;10(11):2824. doi: 10.3390/cells10112824 (PMC8616525; doi:10.3390/cells10112824)
Supplement: Supplementary file 1 [file cells-10-02824-s001.zip › cells-1386423-supplementary.pdf]

Supplementary Materials:

Material and Methods

Determination of label free-cells

To determine if cells after TACS® separation are label free, FICOLL separated PBMCs ($4x10^5$) were stained with 50 µl CD3 specific Strep-tagged Fab-fragments (CD3 Fab; 1:10; 6-8001-0690; IBA GmbH, Göttingen, Germany) for 10 min at RT. After 2 washing steps with 2% BSA in PBS, cells were stained with 50 µl Strep-Tactin APC (1:25; 6-5010-001; IBA GmbH, Göttingen, Germany) for 10 min at RT followed by 2 washing steps with 2% BSA in PBS. This staining was completed by incubating the cells with aCD45-PerCP (1:20; 2D1; BioLegend, San Diego, CA, USA) and aCD3-V450 (1:20; UCHT1; Becton Dickinson, Heidelberg, Germany) for 10 min. To analyze Fab dissociation from the cell surface, stained cells were incubated twice for 7 min with 100 µl 1 mM D-biotin solution (6-6996-001; IBA GmbH, Göttingen, Germany). After two washing steps (2% BSA in PBS), cells were probed again with 50 µl Strep-Tactin APC (1:25; 6-5010-001; IBA GmbH, Göttingen, Germany) for 10 min at RT.

The determination of label free-cells after REAlease® separation followed a similar procedure. FICOLL separated PBMCs ($4x10^5$ cells) were stained with 50 µl CD3-Biotin (1:10; 130-117-038; Miltenyi Biotech) for 10 min at RT. After 2 washing steps with 2% BSA in PBS, cells were stained with 50 µl anti-Biotin APC (1:25; 130-110-952; Miltenyi Biotech, Bergisch Gladbach, Germany) for 10 min at RT followed by 2 washing steps with 2% FCS in PBS. This staining was completed by incubation of cells with aCD45-PerCP (1:20; 2D1; BioLegend, San Diego, CA, USA) and aCD3-V450 (1:20; UCHT1; Becton Dickinson, Heidelberg, Germany) for 10 min. To analyze dissociation of the CD3-Biotin from the cell surface, stained cells were incubated for 7 min with 100 µl Release Bead solution (1:50; 130-117-038; Miltenyi Biotech, Bergisch Gladbach, Germany) followed by incubation for 7 min with 100 µl Release Reagent (1:50; 130-117-038; Miltenyi Biotech, Bergisch Gladbach, Germany) at RT. After two washing steps (2% BSA in PBS), cells were probed again with 50 µl anti-Biotin APC (1:25; 130-110-952; Miltenyi Biotech, Bergisch Gladbach, Germany) for 10 min at RT.

Flow cytometry analyses were performed by a FACSCanto II flow cytometer (Becton Dickinson, Heidelberg, Germany). Data were analysed by the FACS Diva software 8.0.1 (Becton Dickinson, Heidelberg, Germany).

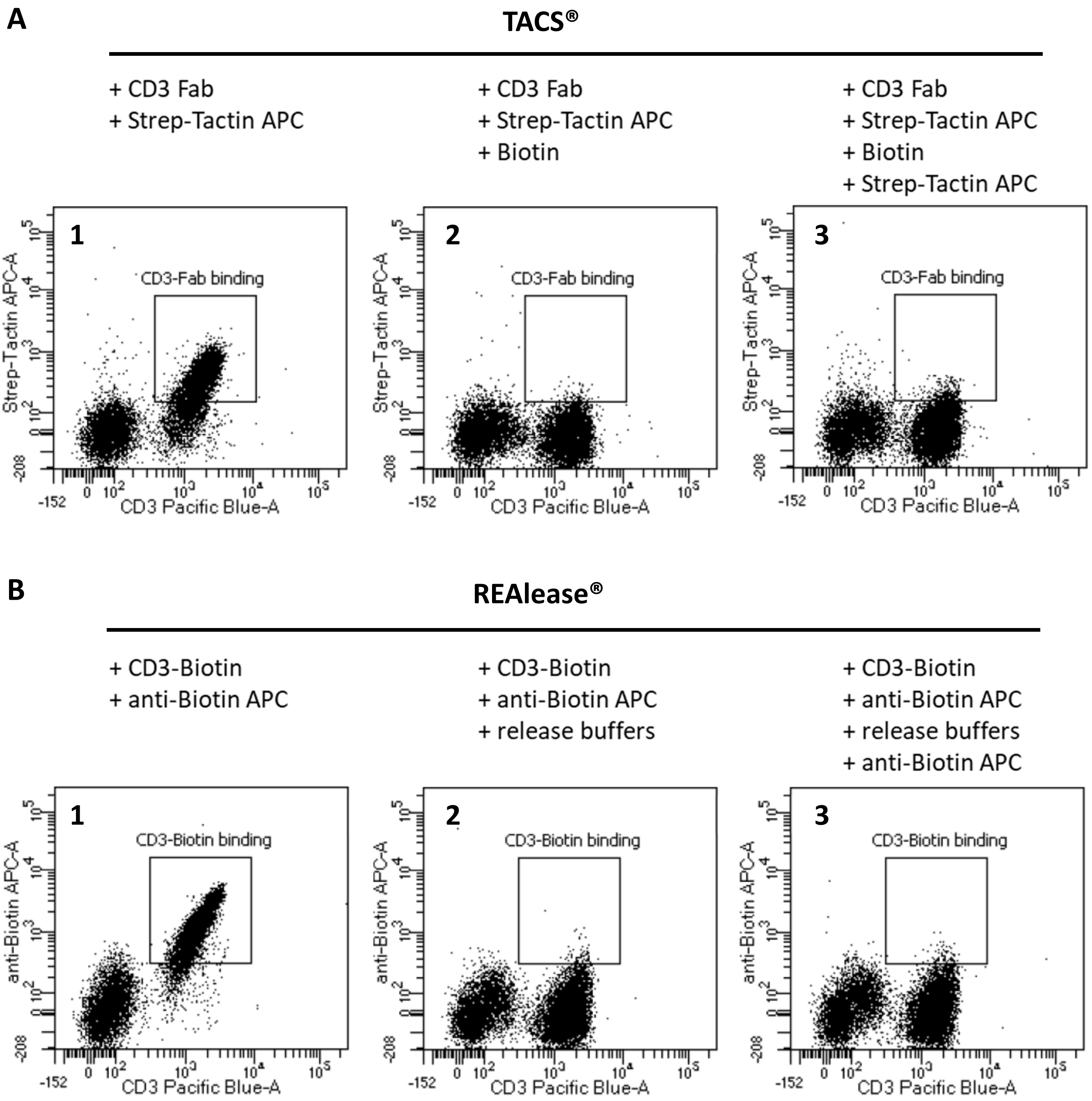

Supplementary Figure S1. Determination of label free-cells.
To test if cells after (A) TACS® and (B) REAlease® separation are label free, PBMCs were stained with CD3-V450 antibody (CD3 Pacific-blue) and CD3 specific Strep-tagged Fab-fragments (CD3 Fab) together with Strep-Tactin APC (ST-APC) (A) or CD3-Biotin together with anti-Biotin APC (B), respectively. Cells were analyzed before (A1 and B1) and after addition of Biotin (A2) or the release buffers (B2). After washing, the cells were probed again with Strep-Tactin APC (A3) or anti-Biotin APC (B3) to visualize if cells are label free.

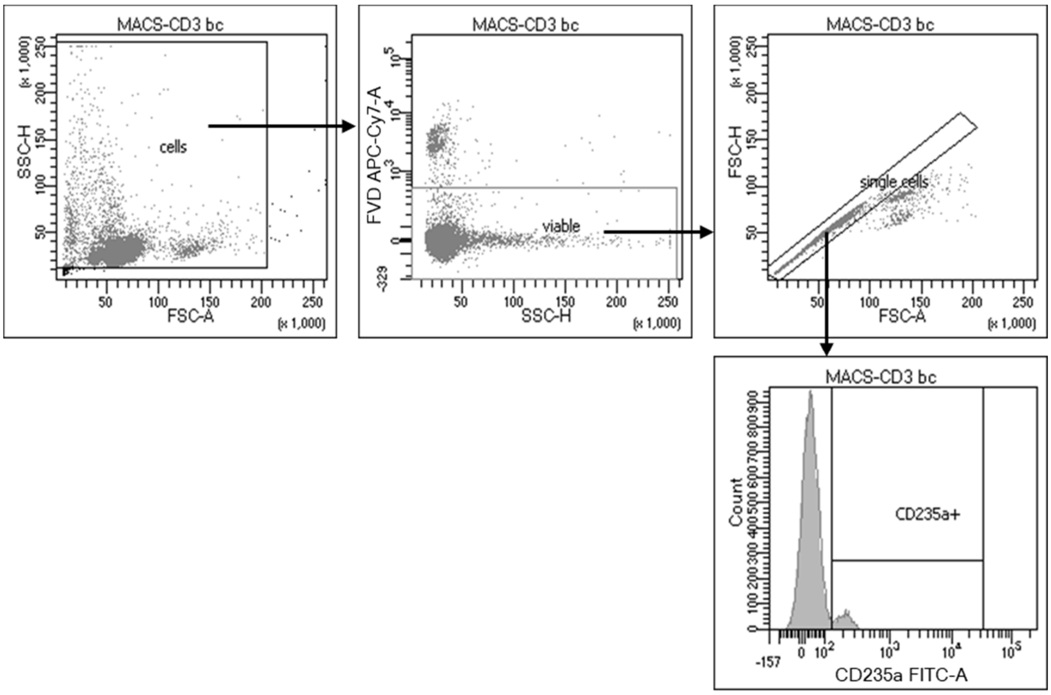

Supplementary Figure S2. Gating strategy to determine erythrocyte contamination.

Cells were first gated on size (FCS-A/SSC-H) followed by exclusion of dead cells (FVD eFluor® 780 (= APC-Cy7-A) positive cells). Subsequently, viable cells were gated on FSC-A/FSC-H to identify single cells followed by determination of erythrocytes by CD235a (FITC) expression.

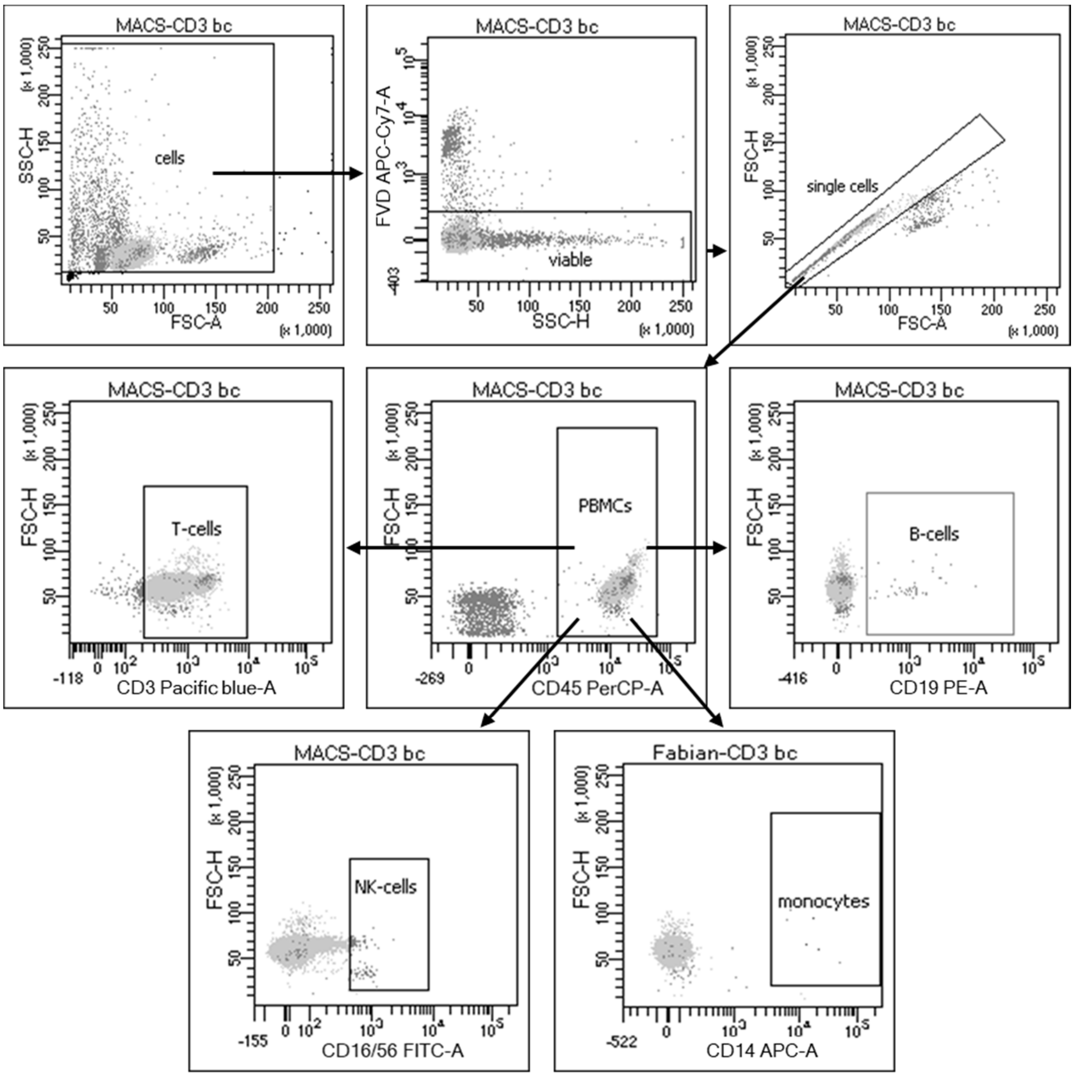

Supplementary Figure S3. Gating strategy to determine the mononuclear white cell population.

Cells were first gated on size (FCS-A/SSC-H), followed by exclusion of dead cells (FVD eFluor® 780 (= APC-Cy7-A) positive cells). Subsequently, viable cells were gated on (FSC-A/FSC-H) to identify single cells, followed by determination of PBMCs via CD45 (PerCP-A) expression. Finally, PBMCs were gated for expression of CD3-Pacific Blue (V450) (T-cells), CD19-PE (B-cells), CD14-APC (monocytes) and CD16/56-FITC (NK-cells).

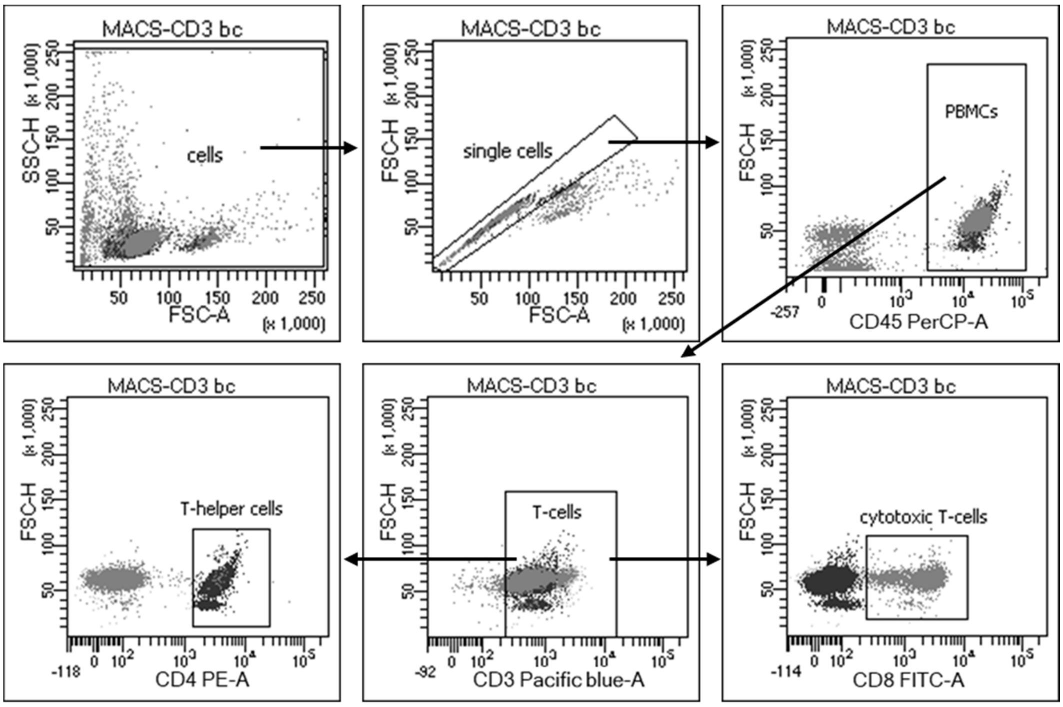

Supplementary Figure S4. Gating strategy to determine the CD4 and CD8 T cell subsets.

Cells were first gated to determine single cells (FSC-A/FSC-H), followed by determination of PBMCs via CD45 (PerCP-A) expression. Subsequently, viable PBMCs were gated for expression of CD3-Pacific Blue (V450) (T-cells) followed by finally gating of T-helper cells by CD4 (PE) and cytotoxic T cells by CD8 (FITC).

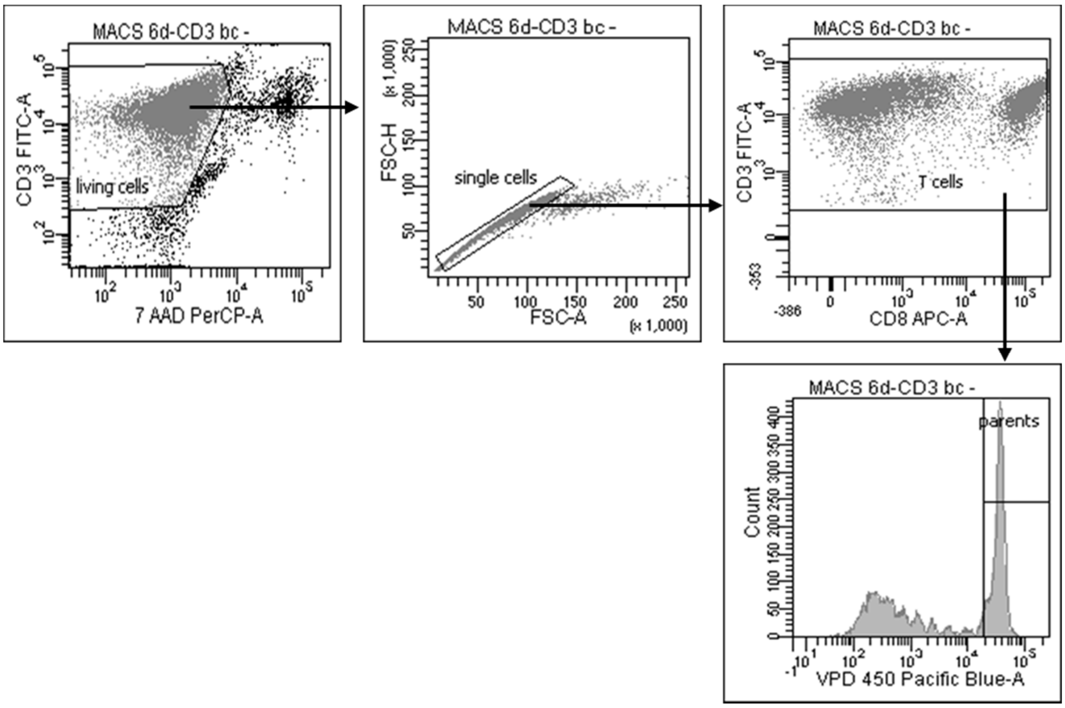

Supplementary Figure S5. Gating strategy to determine proliferation of T cells.

Cells were first gated to determine CD3-positive living (CD3 FITC positive/7-AAD PerCP negative) cells, followed by gating of single cells (FSC-A/FSC-H). Finally, proliferation of CD3 positive cells (CD3 FITC-A) was determined by measuring the VPD450 intensity (VPD450 Pacific-Blue-A).
